# Stability Analysis of a Fractional-Order African Swine Fever Model with Saturation Incidence

**DOI:** 10.3390/ani14131929

**Published:** 2024-06-29

**Authors:** Ruiqing Shi, Yihong Zhang

**Affiliations:** School of Mathematics and Computer Science, Shanxi Normal University, Taiyuan 030031, China; zhangyihong98@163.com

**Keywords:** African Swine Fever, fractional order, saturation incidence, basic reproduction number, stability

## Abstract

**Simple Summary:**

African swine fever is an acute pig disease caused by a highly contagious virus, and so far no cure has been found. Killing live pigs in affected areas is still the most effective way to prevent the spread of the disease. However, this approach can have a devastating impact on a country’s pig industry. Thus, it is necessary to implement strict biosafety prevention and control before the disease begins to spread. In order to further understand the transmission characteristics of the disease and develop effective prevention and control measures, a fractional-order African Swine Fever model with saturation incidence is constructed in this paper. This model, as an effective method to describe the laws of the objective world, is suitable for analyzing the problem of the continued spread or regression of diseases in areas where there are African Swine Fever outbreaks in the real world. Both theoretical analysis and numerical simulations show that timely and effective disinfection measures on pig farms are important to prevent the spread of the disease.

**Abstract:**

This article proposes and analyzes a fractional-order African Swine Fever model with saturation incidence. Firstly, the existence and uniqueness of a positive solution is proven. Secondly, the basic reproduction number and the sufficient conditions for the existence of two equilibriums are obtained. Thirdly, the local and global stability of disease-free equilibrium is studied using the LaSalle invariance principle. Next, some numerical simulations are conducted based on the Adams-type predictor–corrector method to verify the theoretical results, and sensitivity analysis is performed on some parameters. Finally, discussions and conclusions are presented. The theoretical results show that the value of the fractional derivative α will affect both the coordinates of the equilibriums and the speed at which the equilibriums move towards stabilization. When the value of α becomes larger or smaller, the stability of the equilibriums will be changed, which shows the difference between the fractional-order systems and the classical integer-order system.

## 1. Introduction

African Swine Fever (ASF) is an acute and highly contagious viral disease caused by a viral strain of swine fever [1]. The disease has a short onset process and is usually transmitted in domestic and wild pigs. Clinical manifestations include fever, skin cyanosis, significant bleeding in lymph nodes and kidneys, and gastrointestinal mucosa [2]. ASF strains with different virulence have different effects on diseased pigs [3]. Some strains of ASF can lead to almost 100% mortality [4]. Since the first report of ASF in Kenya in 1921, the disease has been widely spread around the world, causing huge losses to pig farming in different countries [5]. The most typical example is Denmark, which is known as the world’s largest exporter of pork. After ASF spread to Denmark, it almost caused a devastating blow to Denmark’s pig farming industry, resulting in serious economic losses for the country [6]. Due to its serious consequences and economic losses, ASF has been included in the Land Animal Health Code by the World Health Organization [7], and many experts have also studied the disease.

Due to the diverse genetic types, large numbers, and complex immune escape mechanisms of ASFV, the development of vaccines targeting ASF is facing difficulties. So far, no effective drugs have been found to prevent and treat this disease. At present, improving comprehensive prevention and control measures for ASF remains the main direction for controlling the spread of the disease. Research has shown that ASF can be transmitted through other organisms, such as infected ticks and mice [8]. It can also be transmitted through direct and indirect contact with infected pigs and contaminated environments [2]. Usually, vehicles and personnel in pig farms carry the virus when passing through epidemic areas. When disinfection is not thorough, this can become a source of transmission. Therefore, strict disinfection and isolation measures for staff and the environment of pig houses are necessary for disease prevention and control. In addition, attention should be paid to the source of pigs and the safety of feed in pig farms to avoid swill feeding.

A series of mathematical models have been established to simulate and study how to better control the spread of ASF. Barongo et al. designed a mathematical model of ASF in 2016 to explore the impact of implementing different strategies over time on disease-related mortality rates [9]. In 2018, Iglesis et al. studied the important role of wild pigs in the spread of ASF [10]. In 2020, Zhang et al. proposed a toy model to explore the transmission mechanism and control strategies of ASF in large-scale pig farms, emphasizing the necessity of disinfection and isolation measures [11]. Shi et al. proposed a fractional-order optimization control model for ASF, using the Hamiltonian function and Pontryagin’s Maximum Principle to find the best strategy to reduce the spread of ASF [12]. In 2021, Kouidere et al. established an ASF model with tick transmission [13], indicating that vector transmission is an important factor concerning ASF infection, and the impact of three biosecurity measures on disease control was explored using optimal control theory. In 2023, Song et al. established an ASF model with asymptomatic infections and infections from other sources of pollution and considered the importance of culling measures for effectively controlling disease spread [14]. These works indicate that strict biosecurity measures are essential for the control of ASF.

Differential equations are a commonly used tool in the field of mathematical modeling, but for some analyses with time memory [15,16,17], integer-order differential equations often cannot be well interpreted. As is well known, the immune system has a memory function. When a specific pathogen invades the body, it will produce memory lymphocytes or antibodies, which are equivalent to the same pathogen invading the body again. Acquired immunity will quickly play a role in preventing infection. Combining the unique memory advantage of fractional-order systems, the modeling method of this equation has been increasingly favored by scholars in recent years [18,19,20]. Due to the fact that the initial values of fractional differential equations defined by Caputo have the same meaning as integer orders, it can solve the problem of it being difficult to find the initial values of traditional fractional-order systems. This advantage has led to the increasing application of fractional-order systems defined by Caputo in fields such as optics and epidemiology [21,22,23,24]. Considering that when the number of susceptible pigs is large and the contact ability between a diseased pig and other healthy pigs is always limited, it is unreasonable to assume that the infectivity is directly proportional to the susceptible pigs. Due to the limitation of contact ability, the infectivity always reaches a saturation state. Therefore, studying infectious disease models with saturation incidence rates has certain practical significance. In summary, this article will use a fractional order system with saturation incidence to analyze the propagation dynamics of ASF. Compared with previous work, this model takes into account the memory function of the immune system and is more consistent with the actual situation.

## 2. Model Formulation

Motivated by [12,14], we will establish a fractional-order ASF model with saturation incidence as below,

(1) $$\left\{\begin{array}{c} \;D^{\alpha }S\left(t\right)=\Lambda -\frac{\beta_{1}S\left(t\right)I\left(t\right)}{1+b_{1}I\left(t\right)}-\beta_{2}S\left(t\right)M\left(t\right)-\mu S\left(t\right)+\delta R\left(t\right),\\ \;D^{\alpha }E\left(t\right)=\frac{\beta_{1}S\left(t\right)I\left(t\right)}{1+b_{1}I\left(t\right)}+\beta_{2}S\left(t\right)M\left(t\right)-\left(\omega +\mu \right)E\left(t\right),\\ \;D^{\alpha }I\left(t\right)=\omega E\left(t\right)-\varepsilon I\left(t\right)-\left(\mu +d\right)I\left(t\right),\\ \;D^{\alpha }M\left(t\right)=hdI\left(t\right)-\varphi M\left(t\right),\\ \;D^{\alpha }R\left(t\right)=\varepsilon I\left(t\right)-\left(\mu +\delta \right)R\left(t\right), \end{array}\right.$$

with initial conditions

S(0),E(0),I(0),M(0),R(0)≥0.

Here, α∈(0,1] and $D^{\alpha }$ is the Caputo fractional-order derivative. The total pig population is given by N(t)=S(t)+E(t)+I(t)+R(t). The biological meanings of state variables and parameters are shown in Table 1.

System (1) is obtained by directly replacing integer orders with fractional derivatives, so there may be an asymmetry in the left and right dimensions of the system. For example, the left side of System (1) has dimension ${\left(\mathrm{time}\right)}^{-\alpha }$, while the right side has dimension ${\left(\mathrm{time}\right)}^{-1}$. We will use the method in [25,26] to modify this issue. The correct form of the modified System (1) will be changed to the following System (2).

(2) $$\left\{\begin{array}{c} \;D^{\alpha }S\left(t\right)=\Lambda^{\alpha }-\frac{\beta_{1}^{\alpha }S\left(t\right)I\left(t\right)}{1+b_{1}I\left(t\right)}-\beta_{2}^{\alpha }S\left(t\right)M\left(t\right)-\mu^{\alpha }S\left(t\right)+\delta^{\alpha }R\left(t\right),\\ \;D^{\alpha }E\left(t\right)=\frac{\beta_{1}^{\alpha }S\left(t\right)I\left(t\right)}{1+b_{1}I\left(t\right)}+\beta_{2}^{\alpha }S\left(t\right)M\left(t\right)-\left(\omega^{\alpha }+\mu^{\alpha }\right)E\left(t\right),\\ \;D^{\alpha }I\left(t\right)=\omega^{\alpha }E\left(t\right)-\varepsilon^{\alpha }I\left(t\right)-\left(\mu^{\alpha }+d^{\alpha }\right)I\left(t\right),\\ \;D^{\alpha }M\left(t\right)=h^{\alpha }d^{\alpha }I\left(t\right)-\varphi^{\alpha }M\left(t\right),\\ \;D^{\alpha }R\left(t\right)=\varepsilon^{\alpha }I\left(t\right)-\left(\mu^{\alpha }+\delta^{\alpha }\right)R\left(t\right). \end{array}\right.$$

Next, we will analyze System (2).

**Table 1:** The biologica meanings of the variables and parameters for System (1).

Variables	Description		
*S*	The density of susceptible pigs		
*E*	The density of exposed pigs		
*I*	The density of infected pigs		
*M*	The density of virus in contaminated items		
*R*	The density of recovered pigs		
Parameters	Description	Value	Refs
Λ	The recruitment rate of pigs	[1.12,1.35]	[12,27]
$\beta_{1}$	ASFV transmission rate with direct contact of infectious pigs	[0.0017, 0.017]	[27]
$\beta_{2}$	Virus transmission rate in contaminated items	[0.0003, 0.0017]	Assumed
$b_{1}$	The saturation constant	[0.003, 0.5]	Assumed
μ	The natural death rate of pigs	[0.0025, 0.0045]	[11,12,14]
δ	The rate of the recovered pigs who become susceptible	[0.02, 0.4243]	[12,27]
ω	The average rate at which an individual passes through the incubation period	[0.12, 0.667]	[12,27]
ε	The rate of the infected pigs who recover	[0.018, 0.418]	Assumed
*d*	The death rate due to the disease	0.27	[11,12]
*h*	The release rate of virus from symptomatic infectious pigs	[0.18, 0.38]	Assumed
φ	The clearance rate of virus	[0.026, 0.126]	[14]

## 3. Qualitatively Analysis of System (2)

### 3.1. The Existence and Uniqueness of Positive Solution for System (2)

It is necessary to prove that the solution of System (2) is positive and bounded in order to make the model biologically meaningful.

Denote

$$\Gamma =\left\{\left(S,E,I,M,R\right)\in R_{+}^{5}:0\leq S+E+I+R\leq \frac{\Lambda^{\alpha }}{\mu^{\alpha }},\;0\leq M\leq \frac{h^{\alpha }d^{\alpha }\Lambda^{\alpha }}{\mu^{\alpha }\varphi^{\alpha }}\right\}.$$

**Theorem** **1.** *System (2) with any positive initial value has a unique solution and* Γ *is positively invariant for System (2).*

**Proof** **of** **Theorem** **1.** Firstly, we will prove that the solution of System (2) with any positive initial value is always non-negative and bounded. Based on System (2), we have

$$\begin{array}{ccc} D^{\alpha }{S|}_{S=0} & = & \Lambda^{\alpha }+\delta^{\alpha }R>0,\;\\ D^{\alpha }{E|}_{E=0} & = & \frac{\beta_{1}^{\alpha }SI}{1+b_{1}I}+\beta_{2}^{\alpha }SM\geq 0,\;\\ D^{\alpha }{I|}_{I=0} & = & \omega^{\alpha }E\geq 0,\;\\ D^{\alpha }{M|}_{M=0} & = & h^{\alpha }d^{\alpha }I\geq 0,\;\\ D^{\alpha }{R|}_{R=0} & = & \varepsilon^{\alpha }I\geq 0. \end{array}$$

Observe the second equation above and combine it with Theorem 2.1 in [28]; we have S(t),E(t),I(t), M(t),R(t)≥0 for any t≥0.

Adding the first three equations of System (2) to the last equation, we obtain

$$\begin{array}{ccc} D^{\alpha }N\left(t\right) & = & D^{\alpha }\left(S\left(t\right)+E\left(t\right)+I\left(t\right)+R\left(t\right)\right)\\ & = & \Lambda^{\alpha }-\mu^{\alpha }S\left(t\right)-\mu^{\alpha }E\left(t\right)-\mu^{\alpha }I\left(t\right)-d^{\alpha }I\left(t\right)-\mu^{\alpha }R\left(t\right)\\ & \leq & \Lambda^{\alpha }-\mu^{\alpha }N\left(t\right), \end{array}$$

which implies that

$$N\left(t\right)\leq \left[-\frac{\Lambda^{\alpha }}{\mu^{\alpha }}+N\left(0\right)\right]E_{\alpha }\left(-\mu^{\alpha }t^{\alpha }\right)+\frac{\Lambda^{\alpha }}{\mu^{\alpha }}.$$

Since $E_{\alpha }\left(-\mu^{\alpha }t^{\alpha }\right)\geq 0$ for t≥0, thus

$$N\left(t\right)\leq \frac{\Lambda^{\alpha }}{\mu^{\alpha }},\;\forall t\geq 0,$$

provided that $N\left(0\right)\leq \frac{\Lambda^{\alpha }}{\mu^{\alpha }}$.

From the above equation, it is easy to know that $I\left(t\right)\leq \frac{\Lambda^{\alpha }}{\mu^{\alpha }}$, combined with the fourth equation of System (2), we will obtain

$$\begin{array}{ccc} D^{\alpha }M\left(t\right) & = & h^{\alpha }d^{\alpha }I\left(t\right)-\varphi^{\alpha }M\left(t\right)\;\\ & \leq & \frac{h^{\alpha }d^{\alpha }\Lambda^{\alpha }}{\mu^{\alpha }}-\varphi^{\alpha }M\left(t\right), \end{array}$$

which implies that

$$M\left(t\right)\leq \left[-\frac{h^{\alpha }d^{\alpha }\Lambda^{\alpha }}{\mu^{\alpha }\varphi^{\alpha }}+M\left(0\right)\right]E_{\alpha }\left(-\varphi^{\alpha }t^{\alpha }\right)+\frac{h^{\alpha }d^{\alpha }\Lambda^{\alpha }}{\mu^{\alpha }\varphi^{\alpha }}.$$

Since $E_{\alpha }\left(-\varphi^{\alpha }t^{\alpha }\right)\geq 0$ for t≥0, thus

$$M\left(t\right)\leq \frac{h^{\alpha }d^{\alpha }\Lambda^{\alpha }}{\mu^{\alpha }\varphi^{\alpha }},\;\forall t\geq 0,$$

provided that $M\left(0\right)\leq \frac{h^{\alpha }d^{\alpha }\Lambda^{\alpha }}{\mu^{\alpha }\varphi^{\alpha }}.$

Therefore, S(t),E(t),I(t),M(t),R(t)≥0, and $N\left(t\right)\leq \frac{\Lambda^{\alpha }}{\mu^{\alpha }},$ $M\left(t\right)\leq \frac{h^{\alpha }d^{\alpha }\Lambda^{\alpha }}{\mu^{\alpha }\varphi^{\alpha }}$, ∀t≥0, which means that Γ is positively invariant for System (2).

Secondly, we will prove that System (2) with any positive initial value has a unique solution.

Denote the right side of System (2) as vector function $f(t,\vec{x}\left(t\right))$, and

$$\begin{array}{c} \vec{x}\left(t\right)=\left(\begin{array}{c} x_{1}\left(t\right)\\ x_{2}\left(t\right)\\ x_{3}\left(t\right)\\ x_{4}\left(t\right)\\ x_{5}\left(t\right) \end{array}\right),\;A_{1}=\left(\begin{array}{ccccc} -\mu^{\alpha } & 0 & 0 & 0 & \delta^{\alpha }\\ 0 & -(\omega^{\alpha }+\mu^{\alpha }) & 0 & 0 & 0\\ 0 & \omega^{\alpha } & -(\varepsilon^{\alpha }+\mu^{\alpha }+d^{\alpha }) & 0 & 0\\ 0 & 0 & h^{\alpha }d^{\alpha } & -\varphi^{\alpha } & 0\\ 0 & 0 & \varepsilon^{\alpha } & 0 & -(\mu^{\alpha }+\delta^{\alpha }) \end{array}\right),\\ \xi =\left(\begin{array}{c} \Lambda^{\alpha }\\ 0\\ 0\\ 0\\ 0 \end{array}\right),\;A_{2}=\left(\begin{array}{ccccc} 0 & 0 & 0 & -\beta_{2} & 0\\ 0 & 0 & 0 & \beta_{2} & 0\\ 0 & 0 & 0 & 0 & 0\\ 0 & 0 & 0 & 0 & 0\\ 0 & 0 & 0 & 0 & 0 \end{array}\right),\;A_{3}=\left(\begin{array}{ccccc} -\beta_{1} & 0 & 0 & 0 & 0\\ \beta_{1} & 0 & 0 & 0 & 0\\ 0 & 0 & 0 & 0 & 0\\ 0 & 0 & 0 & 0 & 0\\ 0 & 0 & 0 & 0 & 0 \end{array}\right), \end{array}$$

where $x_{1}\left(t\right)=S\left(t\right),x_{2}\left(t\right)=E\left(t\right),x_{3}\left(t\right)=I\left(t\right),x_{4}\left(t\right)=M\left(t\right),x_{5}\left(t\right)=R\left(t\right),x_{1}\left(0\right)=S\left(0\right)$, $x_{2}\left(0\right)=E\left(0\right),x_{3}\left(0\right)=I\left(0\right),x_{4}\left(0\right)=M\left(0\right),x_{5}\left(0\right)=R\left(0\right)$. Then, System (2) can be written as

$$\begin{array}{ccc} \;D^{\alpha }\vec{x}\left(t\right) & = & A_{1}\vec{x}\left(t\right)+x_{1}\left(t\right)A_{2}\vec{x}\left(t\right)+\frac{x_{3}\left(t\right)}{1+b_{1}x_{3}\left(t\right)}A_{3}\vec{x}\left(t\right)+\xi \;\\ & \leq & \left(∥A_{1}∥+∥x_{1}\left(t\right)A_{2}∥+∥\frac{x_{3}\left(t\right)}{1+b_{1}x_{3}\left(t\right)}∥\cdot ∥A_{3}∥\right)\vec{x}\left(t\right)+∥\xi ∥\;\\ & \leq & \left(∥A_{1}∥+\frac{\Lambda^{\alpha }}{\mu^{\alpha }}∥A_{2}∥+\frac{1}{b_{1}}∥A_{3}∥\right)\vec{x}\left(t\right)+∥\xi ∥\;\\ & ≐ & \theta_{1}\vec{x}\left(t\right)+\theta_{2}. \end{array}$$

Thus, the fourth condition of Theorem 3.1 in [28] is also satisfied for System (2). According to that theorem, we know that System (2) has a unique positive solution for any positive initial value. This completes the proof. □

### 3.2. Basic Reproduction Number and the Existence of Equilibriums

For all infectious disease models, the basic reproductive number $R_{0}$ is an important indicator for predicting disease development trends [29]. By using the method of the next generation matrix [30], the basic reproduction number of System (2) is derived as follows:

$$\begin{array}{ccc} R_{0} & = & \rho (FV^{-1})\\ & = & \frac{\beta_{1}^{\alpha }\Lambda^{\alpha }\omega^{\alpha }}{2\mu^{\alpha }\left(\omega^{\alpha }+\mu^{\alpha }\right)\left(\varepsilon^{\alpha }+\mu^{\alpha }+d^{\alpha }\right)}\;\\ & & +\frac{1}{2}\sqrt{{\left(\frac{\beta_{1}^{\alpha }\Lambda^{\alpha }\omega^{\alpha }}{\mu^{\alpha }\left(\omega^{\alpha }+\mu^{\alpha }\right)\left(\varepsilon^{\alpha }+\mu^{\alpha }+d^{\alpha }\right)}\right)}^{2}+4\frac{\beta_{2}^{\alpha }\Lambda^{\alpha }h^{\alpha }d^{\alpha }\omega^{\alpha }}{\varphi^{\alpha }\mu^{\alpha }\left(\omega^{\alpha }+\mu^{\alpha }\right)\left(\varepsilon^{\alpha }+\mu^{\alpha }+d^{\alpha }\right)}}, \end{array}$$

where $\rho (FV^{-1})$ represents the spectral radius of matrix $FV^{-1}$, and

$$F=\left(\begin{array}{ccc} \;0 & \frac{\beta_{1}\Lambda^{\alpha }}{\mu^{\alpha }} & \frac{\beta_{2}\Lambda^{\alpha }}{\mu^{\alpha }}\\ \;0 & 0 & 0\\ \;0 & h^{\alpha }d^{\alpha } & 0 \end{array}\right),\;V=\left(\begin{array}{ccc} \omega^{\alpha }+\mu^{\alpha } & 0 & 0\\ \;-\omega^{\alpha } & \varepsilon^{\alpha }+\mu^{\alpha }+d^{\alpha } & 0\\ \;0 & 0 & \varphi^{\alpha } \end{array}\right).$$

Define

$$R_{c}=\frac{\beta_{1}^{\alpha }\Lambda^{\alpha }\omega^{\alpha }}{\mu^{\alpha }\left(\omega^{\alpha }+\mu^{\alpha }\right)\left(\varepsilon^{\alpha }+\mu^{\alpha }+d^{\alpha }\right)}+\frac{\beta_{2}^{\alpha }\Lambda^{\alpha }h^{\alpha }d^{\alpha }\omega^{\alpha }}{\varphi^{\alpha }\mu^{\alpha }\left(\omega^{\alpha }+\mu^{\alpha }\right)\left(\varepsilon^{\alpha }+\mu^{\alpha }+d^{\alpha }\right)}.$$

**Remark** **1.** *It is easy to verify that*

*(i)*    *If $R_{c}<1,$ then $R_{0}<1$; if $R_{c}>1,$ then $R_{0}>1$;*
*(ii)*  *If $R_{0}<1,$ then $R_{c}<1$; if $R_{0}>1,$ then $R_{c}>1$.*

In order to obtain the equilibriums of System (2), let the right side of Equation (2) equal zero; we can get the following algebraic equations:

(3) $$\left\{\begin{array}{c} \;\Lambda^{\alpha }-\frac{\beta_{1}^{\alpha }SI}{1+b_{1}I}-\beta_{2}^{\alpha }SM-\mu^{\alpha }S+\delta^{\alpha }R=0,\\ \;\frac{\beta_{1}^{\alpha }SI}{1+b_{1}I}+\beta_{2}^{\alpha }SM-\left(\omega^{\alpha }+\mu^{\alpha }\right)E=0,\\ \;\omega^{\alpha }E-\varepsilon^{\alpha }I-\left(\mu^{\alpha }+d^{\alpha }\right)I=0,\\ \;h^{\alpha }d^{\alpha }I-\varphi^{\alpha }M=0,\\ \;\varepsilon^{\alpha }I-\left(\mu^{\alpha }+\delta^{\alpha }\right)R=0. \end{array}\right.$$

After a simple calculation, we can show that System (2) always exists a disease-free equilibrium $E_{0}=\left(\frac{\Lambda^{\alpha }}{\mu^{\alpha }},0,0,0,0\right)$. Denote the positive solution of Equation (3) as $E_{1}=\left(S^{*},E^{*},I^{*},M^{*},R^{*}\right)$; then, we have

$$R^{*}=\frac{\varepsilon^{\alpha }}{\mu^{\alpha }+\delta^{\alpha }}I^{*},\;M^{*}=\frac{h^{\alpha }d^{\alpha }}{\varphi^{\alpha }}I^{*},\;E^{*}=\frac{\varepsilon^{\alpha }+\mu^{\alpha }+d^{\alpha }}{\omega^{\alpha }}I^{*},$$

$$S^{*}=\frac{\Lambda^{\alpha }\left(\mu^{\alpha }+\delta^{\alpha }\right)\omega^{\alpha }+\delta^{\alpha }\varepsilon^{\alpha }\omega^{\alpha }I^{*}-\left(\omega^{\alpha }+\mu^{\alpha }\right)\left(\varepsilon^{\alpha }+\mu^{\alpha }+d^{\alpha }\right)\left(\mu^{\alpha }+\delta^{\alpha }\right)I^{*}}{\mu^{\alpha }\left(\mu^{\alpha }+\delta^{\alpha }\right)\omega^{\alpha }},$$

and $I^{*}$ is the positive root of the following equation

(4) $$a_{1}I^{2}+a_{2}I+a_{3}=0,$$

where

(5) $$\begin{array}{lll} a_{1} & = & -\frac{b_{1}d^{\alpha }h^{\alpha }}{\varphi^{\alpha }}\left\{\beta_{2}^{\alpha }\delta^{\alpha }\varepsilon^{\alpha }\mu^{\alpha }+\beta_{2}\left(\mu^{\alpha }+\omega^{\alpha }\right)\left[\left(d^{\alpha }+\mu^{\alpha }\right)\left(\delta^{\alpha }+\mu^{\alpha }\right)+\mu^{\alpha }\varepsilon^{\alpha }\right]\right\},\\ a_{2} & = & -\left(\delta^{\alpha }+\mu^{\alpha }\right)\left(\mu^{\alpha }+\omega^{\alpha }\right)\left(\varepsilon^{\alpha }+\mu^{\alpha }+d^{\alpha }\right)\left(\frac{d^{\alpha }h^{\alpha }}{\varphi^{\alpha }}\beta_{2}+\beta_{1}+b_{1}\mu^{\alpha }\right)\;\\ \; & \; & +\delta^{\alpha }\varepsilon^{\alpha }\omega^{\alpha }\left(\beta_{1}+\frac{d^{\alpha }h^{\alpha }\beta_{2}}{\varphi^{\alpha }}\right)+\frac{d^{\alpha }h^{\alpha }b_{1}\beta_{2}^{\alpha }\Lambda^{\alpha }\omega^{\alpha }\left(\delta^{\alpha }+\mu^{\alpha }\right)}{\varphi^{\alpha }},\;\\ a_{3} & = & \mu^{\alpha }\left(\delta^{\alpha }+\mu^{\alpha }\right)\left(\mu^{\alpha }+\omega^{\alpha }\right)\left(\varepsilon^{\alpha }+\mu^{\alpha }+d^{\alpha }\right)\left(R_{c}-1\right). \end{array}$$

From the above argument, we obtain the following result.

**Theorem** **2.**

 

*(i)*    *For System (2), there always exists a disease-free equilibrium, $E_{0}$.*
*(ii)*  *When $R_{c}>1$, System (2) has a unique endemic equilibrium, $E_{1}=\left(S^{*},E^{*},I^{*},M^{*},R^{*}\right)$.*

### 3.3. Stability of the Disease-Free Equilibrium $E_{0}$

The Jacobian matrix of System (2), evaluated at the disease-free equilibrium, $E_{0}$, is given by

$$J\left(E_{0}\right)=\left(\begin{array}{ccccc} \;\mu^{\alpha } & 0 & -\frac{\beta_{1}^{\alpha }\Lambda^{\alpha }}{\mu^{\alpha }} & -\frac{\beta_{2}^{\alpha }\Lambda^{\alpha }}{\mu^{\alpha }} & \delta^{\alpha }\;\\ 0 & -(\omega^{\alpha }+\mu^{\alpha }) & \frac{\beta_{1}^{\alpha }\Lambda^{\alpha }}{\mu^{\alpha }} & \frac{\beta_{2}^{\alpha }\Lambda^{\alpha }}{\mu^{\alpha }} & 0\;\\ 0 & \omega^{\alpha } & -(\varepsilon^{\alpha }+\mu^{\alpha }+d^{\alpha }) & 0 & 0\;\\ 0 & 0 & h^{\alpha }d^{\alpha } & -\varphi^{\alpha } & 0\;\\ 0 & 0 & \varepsilon^{\alpha } & 0 & -(\mu^{\alpha }+\delta^{\alpha }) \end{array}\right).$$

It is easy to know that two eigenvalues of $J(E_{0})$ is $\lambda_{1}=-\mu^{\alpha }<0$, $\lambda_{2}=-\left(\mu^{\alpha }+\delta^{\alpha }\right)<0$, and the remaining eigenvalues are determined by the following equation:

(6) $$y^{3}+b_{1}y^{2}+b_{2}y+b_{3}=0,$$

where

$$\begin{array}{ccc} b_{1} & = & \varphi^{\alpha }+d^{\alpha }+\varepsilon^{\alpha }+\omega^{\alpha }+2\mu^{\alpha },\\ b_{2} & = & \left(\mu^{\alpha }+\omega^{\alpha }\right)\left(\mu^{\alpha }+d^{\alpha }+\varepsilon^{\alpha }\right)\left(1-R_{c}\right)+\frac{\beta_{2}^{\alpha }d^{\alpha }h^{\alpha }\Lambda^{\alpha }\omega^{\alpha }}{\varphi^{\alpha }\mu^{\alpha }}+\varphi^{\alpha }\left(2\mu^{\alpha }+d^{\alpha }+\varepsilon^{\alpha }+\omega^{\alpha }\right),\\ b_{3} & = & \varphi^{\alpha }\left(\mu^{\alpha }+\omega^{\alpha }\right)\left(\mu^{\alpha }+d^{\alpha }+\varepsilon^{\alpha }\right)\left(1-R_{c}\right). \end{array}$$

If $R_{c}<1$, then $b_{i}>0$, i=1,2,3, and

$$\begin{array}{ccc} \;b_{1}b_{2}-b_{3} & = & \left(2\mu^{\alpha }+\varphi^{\alpha }+d^{\alpha }+\varepsilon^{\alpha }+\omega^{\alpha }\right)[\left(\mu^{\alpha }+\omega^{\alpha }\right)\left(\mu^{\alpha }+d^{\alpha }+\varepsilon^{\alpha }\right)\left(1-R_{c}\right)\;\\ & & +\frac{\beta_{2}^{\alpha }d^{\alpha }h^{\alpha }\Lambda^{\alpha }\omega^{\alpha }}{\varphi^{\alpha }\mu^{\alpha }}+\varphi^{\alpha }\left(2\mu^{\alpha }+d^{\alpha }+\varepsilon^{\alpha }+\omega^{\alpha }\right)]\;\\ & & -\varphi^{\alpha }\left(\mu^{\alpha }+\omega^{\alpha }\right)\left(\mu^{\alpha }+d^{\alpha }+\varepsilon^{\alpha }\right)\left(1-R_{c}\right)\;\\ & = & \left(2\mu^{\alpha }+d^{\alpha }+\varepsilon^{\alpha }+\omega^{\alpha }\right)[\left(\mu^{\alpha }+\omega^{\alpha }\right)\left(\mu^{\alpha }+d^{\alpha }+\varepsilon^{\alpha }\right)\left(1-R_{c}\right)\;\\ & & +\frac{\beta_{2}^{\alpha }d^{\alpha }h^{\alpha }\Lambda^{\alpha }\omega^{\alpha }}{\varphi^{\alpha }\mu^{\alpha }}+\varphi^{\alpha }\left(2\mu^{\alpha }+d^{\alpha }+\varepsilon^{\alpha }+\omega^{\alpha }\right)]\;\\ & & +\frac{\beta_{2}^{\alpha }d^{\alpha }h^{\alpha }\Lambda^{\alpha }\omega^{\alpha }}{\mu^{\alpha }}+\varphi^{2\alpha }\left(2\mu^{\alpha }+d^{\alpha }+\varepsilon^{\alpha }+\omega^{\alpha }\right)\\ & > & 0. \end{array}$$

According to the Routh–Hurwitz criteria [31], we can get the following result.

**Theorem** **3.** *If $R_{c}<1$, then the disease-free equilibrium, $E_{0}$, is locally asymptotically stable.*

Next, we investigate the global dynamics of the disease-free equilibrium.

**Theorem** **4.** *If $R_{c}\leq 1$, then the disease-free equilibrium, $E_{0}$, is global asymptotically stable.*

**Proof** **of** **Theorem** **4.** Consider the following Lyapunov function:

$$V=\omega^{\alpha }E+\left(\omega^{\alpha }+\mu^{\alpha }\right)I.$$

Then, we get the derivative of *V* along the solution of System (2)

$$\begin{array}{ccc} \;D^{\alpha }{V|}_{\left(2\right)} & = & \frac{\omega^{\alpha }\beta_{1}^{\alpha }SI}{1+b_{1}I}+\omega^{\alpha }\beta_{2}^{\alpha }SM-\omega^{\alpha }\left(\omega^{\alpha }+\mu^{\alpha }\right)E\\ \; & & +\omega^{\alpha }\left(\omega^{\alpha }+\mu^{\alpha }\right)E-\left(\omega^{\alpha }+\mu^{\alpha }\right)\left(\varepsilon^{\alpha }+\mu^{\alpha }+d^{\alpha }\right)I\\ \; & \leq & \omega^{\alpha }\beta_{1}^{\alpha }SI+\omega^{\alpha }\beta_{2}^{\alpha }SM-\left(\omega^{\alpha }+\mu^{\alpha }\right)\left(\varepsilon^{\alpha }+\mu^{\alpha }+d^{\alpha }\right)I\\ \; & \leq & \frac{\omega^{\alpha }\beta_{1}^{\alpha }\Lambda^{\alpha }I}{\mu^{\alpha }}+\frac{\omega^{\alpha }\beta_{2}^{\alpha }\Lambda^{\alpha }M}{\mu^{\alpha }}-\left(\omega^{\alpha }+\mu^{\alpha }\right)\left(\varepsilon^{\alpha }+\mu^{\alpha }+d^{\alpha }\right)I\\ \; & \leq & \frac{\omega^{\alpha }\beta_{1}^{\alpha }\Lambda^{2\alpha }}{\mu^{2\alpha }}+\frac{\omega^{\alpha }\beta_{2}^{\alpha }\Lambda^{2\alpha }h^{\alpha }d^{\alpha }}{\mu^{2\alpha }\varphi^{\alpha }}-\left(\omega^{\alpha }+\mu^{\alpha }\right)\left(\varepsilon^{\alpha }+\mu^{\alpha }+d^{\alpha }\right)\frac{\Lambda^{\alpha }}{\mu^{\alpha }}\\ \; & = & \frac{\Lambda^{\alpha }}{\mu^{\alpha }}\left(\omega^{\alpha }+\mu^{\alpha }\right)\left(\varepsilon^{\alpha }+\mu^{\alpha }+d^{\alpha }\right)\left(R_{c}-1\right). \end{array}$$

If $R_{c}\leq 1$, then we obviously have $D^{\alpha }{L|}_{\left(2\right)}\leq 0$. The invariant set of System (2) on the set $\left\{\left(S,E,I,M,R\right)\in \Gamma :D^{\alpha }L{|}_{\left(2\right)}=0\right\}$ is the singleton $\left\{E_{0}\right\}$. According to the LaSalle invariance principle [32], we know that $E_{0}$ is global asymptotically stable.

The proof of this theorem is complete. □

### 3.4. Stability of the Endemic Equilibrium $E_{1}$

The Jacobian matrix of System (2) evaluated at the endemic equilibrium, $E_{1}$, is given by

$$J\left(E_{1}\right)=\left(\begin{array}{ccccc} \;\kappa_{1} & 0 & \kappa_{2} & -\beta_{2}^{\alpha }S^{*} & \delta^{\alpha }\;\\ \kappa_{3} & \kappa_{4} & \kappa_{5} & \beta_{2}^{\alpha }S^{*} & 0\;\\ 0 & \omega^{\alpha } & \kappa_{6} & 0 & 0\;\\ 0 & 0 & h^{\alpha }d^{\alpha } & -\varphi^{\alpha } & 0\;\\ 0 & 0 & \varepsilon^{\alpha } & 0 & \kappa_{7} \end{array}\right),$$

where

$$\kappa_{1}=-\frac{\beta_{1}^{\alpha }I^{*}}{1+b_{1}I^{*}}-\beta_{2}^{\alpha }M^{*}-\mu^{\alpha },\;\kappa_{2}=-\frac{\beta_{1}^{\alpha }S^{*}}{{\left(1+b_{1}I^{*}\right)}^{2}},$$

$$\kappa_{3}=\frac{\beta_{1}^{\alpha }I^{*}}{1+b_{1}I^{*}}+\beta_{2}^{\alpha }M^{*},\;\kappa_{4}=-\left(\omega^{\alpha }+\mu^{\alpha }\right),$$

$$\kappa_{5}=\frac{\beta_{1}^{\alpha }S^{*}}{{\left(1+b_{1}I^{*}\right)}^{2}},\;\kappa_{6}=-\left(\varepsilon^{\alpha }+\mu^{\alpha }+d^{\alpha }\right),\;\kappa_{7}=-\left(\mu^{\alpha }+\delta^{\alpha }\right).$$

By simple calculation, we obtain the corresponding characteristic equation of $J(E_{1})$ as

(7) $$\lambda^{5}+\omega_{1}\lambda^{4}+\omega_{2}\lambda^{3}+\omega_{3}\lambda^{2}+\omega_{4}\lambda +\omega_{5}=0,$$

where

$$\begin{array}{ccc} \omega_{1} & = & \varphi^{\alpha }-\kappa_{1}-\kappa_{4}-\kappa_{6}-\kappa_{7},\;\\ \omega_{2} & = & \kappa_{1}\kappa_{4}-\varphi^{\alpha }\left(\kappa_{4}+\kappa_{6}+\kappa_{7}+\kappa_{1}\right)+\left(\kappa_{1}+\kappa_{4}\right)\left(\kappa_{6}+\kappa_{7}\right)+\kappa_{6}\kappa_{7}-\kappa_{5}\omega^{\alpha },\;\\ \omega_{3} & = & \varphi^{\alpha }\kappa_{1}\left(\kappa_{4}+\kappa_{6}+\kappa_{7}\right)+\left(\varphi^{\alpha }\kappa_{4}-\kappa_{1}\kappa_{4}\right)\left(\kappa_{6}+\kappa_{7}\right)+\varphi^{\alpha }\kappa_{6}\kappa_{7}-\kappa_{6}\kappa_{7}\left(\kappa_{1}+\kappa_{4}\right)\;\\ & & -\varphi^{\alpha }\kappa_{5}\omega^{\alpha }-\kappa_{2}\kappa_{3}\omega^{\alpha }+\kappa_{5}\omega^{\alpha }\left(\kappa_{1}+\kappa_{7}\right)-S^{*}\beta_{2}^{*}d^{\alpha }h^{\alpha }\omega^{\alpha },\;\\ \omega_{4} & = & \kappa_{4}\kappa_{7}\left(\kappa_{1}\kappa_{6}-\varphi^{\alpha }\kappa_{1}\right)-\varphi^{\alpha }\kappa_{6}\kappa_{7}\left(\kappa_{1}+\kappa_{4}\right)-\delta^{\alpha }\varepsilon^{\alpha }\kappa_{3}\omega^{\alpha }-\varphi^{\alpha }\kappa_{1}\kappa_{4}\kappa_{6}\;\\ & & +\left(\kappa_{7}\omega^{\alpha }-\varphi^{\alpha }\omega^{\alpha }\right)\left(\kappa_{2}\kappa_{3}-\kappa_{1}\kappa_{5}\right)+\varphi^{\alpha }\omega^{\alpha }\kappa_{5}\kappa_{7}+S^{*}\beta_{2}^{\alpha }d^{\alpha }h^{\alpha }\omega^{\alpha }\left(\kappa_{1}+\kappa_{3}+\kappa_{7}\right),\;\\ \omega_{5} & = & -\varphi^{\alpha }\delta^{\alpha }\varepsilon^{\alpha }\kappa_{3}\omega^{\alpha }+\varphi^{\alpha }\kappa_{1}\kappa_{4}\kappa_{6}\kappa_{7}+\varphi^{\alpha }\kappa_{7}\omega^{\alpha }\left(\kappa_{2}\kappa_{3}-\kappa_{1}\kappa_{5}\right)-\kappa_{7}\omega^{\alpha }\delta^{\alpha }\beta_{2}^{\alpha }d^{\alpha }h^{\alpha }\left(\kappa_{1}+\kappa_{3}\right). \end{array}$$

Denote

$$H_{1}=\omega_{1},\;H_{2}=\left|\begin{array}{cc} \;\omega_{1} & \omega_{3}\\ 1 & \omega_{2} \end{array}\right|,\;H_{3}=\left|\begin{array}{ccc} \omega_{1} & \omega_{3} & \omega_{5}\\ 1 & \omega_{2} & \omega_{4}\\ 0 & \omega_{1} & \omega_{3} \end{array}\right|,$$

$$H_{4}=\left|\begin{array}{cccc} \omega_{1} & \omega_{3} & \omega_{5} & 0\\ 1 & \omega_{2} & \omega_{4} & 0\\ 0 & \omega_{1} & \omega_{3} & \omega_{5}\\ 0 & 1 & \omega_{2} & \omega_{4} \end{array}\right|,\;H_{5}=\left|\begin{array}{ccccc} \omega_{1} & \omega_{3} & \omega_{5} & 0 & 0\\ 1 & \omega_{2} & \omega_{4} & 0 & 0\\ 0 & \omega_{1} & \omega_{3} & \omega_{5} & 0\\ 0 & 1 & \omega_{2} & \omega_{4} & 0\\ 0 & 0 & \omega_{1} & \omega_{3} & \omega_{5} \end{array}\right|.$$

According to the Routh–Hurwitz criterion [31], we find that if, and only if, the coefficients $\omega_{i}$ satisfy $H_{i}>0$ ( i=1,2,3,4,5), then all roots of Equation (7) have negative real parts.

**Theorem** **5.**

 

*(i)*    *If $H_{i}>0,i=1,\cdots ,5$ then the endemic equilibrium $E_{1}$ is locally asymptotically stable.*
*(ii)*  *When α∈(0,1), according to Lemma 3 in [33], if all roots of Equation* (7) *satisfy $|arg\left(\lambda_{i}\right)|>\frac{\alpha \pi }{2},i=1,\cdots ,5,$ then $E_{1}$ is still locally stable.*

## 4. Examples and Numerical Simulations

In this section, the so-called Adams-type predictor–corrector method and Matlab tool will be used for numerical simulations. In addition, some parameter sensitivity analysis will be conducted. Most of the data in this article are based on the parameter values in [9], the collection of ASF related data from large pig farms in China [11], and the collection of ASF-related data in the Chinese region [14]. As the model in this article has been modified and improved, it is necessary to input the actual parameter values of the local area into the model for debugging in practical application and predict the propagation of ASF in the local area within an acceptable error range.

### Examples and Numerical Simulations for System (2)

**Example** **1.** *Fix the following parameter values: Λ=1.35, $\beta_{1}=0.0017$, $\beta_{2}=0.0003$, μ=0.0045, δ=0.02, $b_{1}=0.007$, ω=0.25, ε=0.3, d=0.27.*

**Figure 1:**
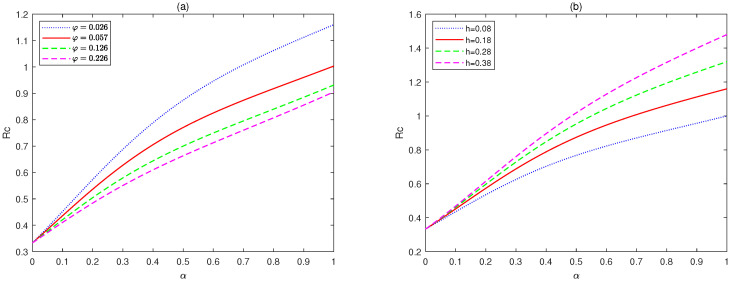
The relationship between $R_{c}$ and the parameter α, (**a**) for different values of φ, (**b**) for different values of *h*.

**Figure 2:**
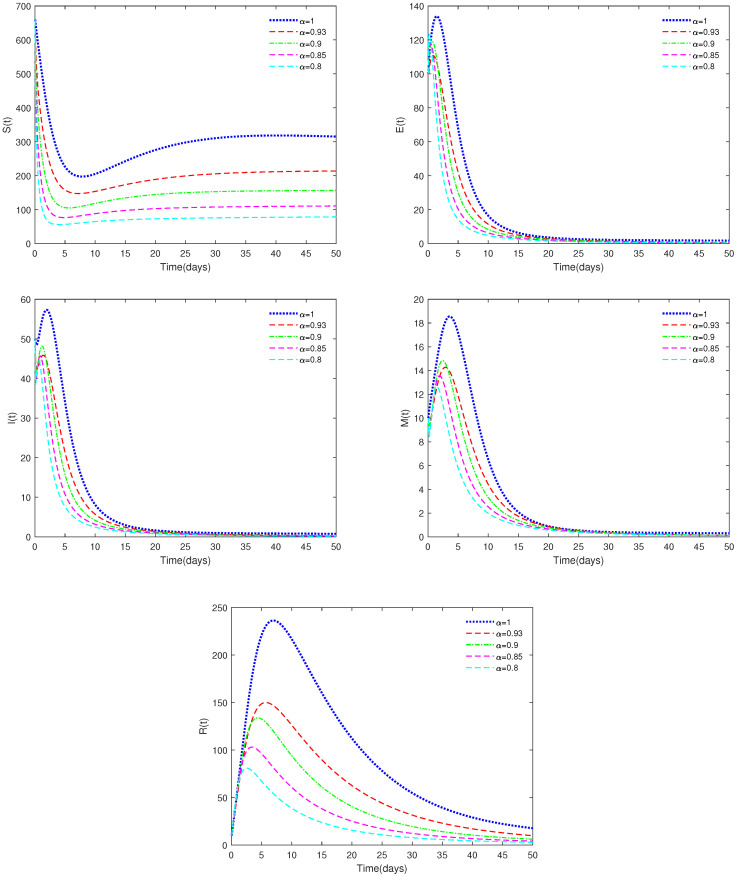
Time series of System (2) for different values of α. Here, $R_{c}\in \left[0.8353,\;0.9314\right]<1$.

**Figure 3:**
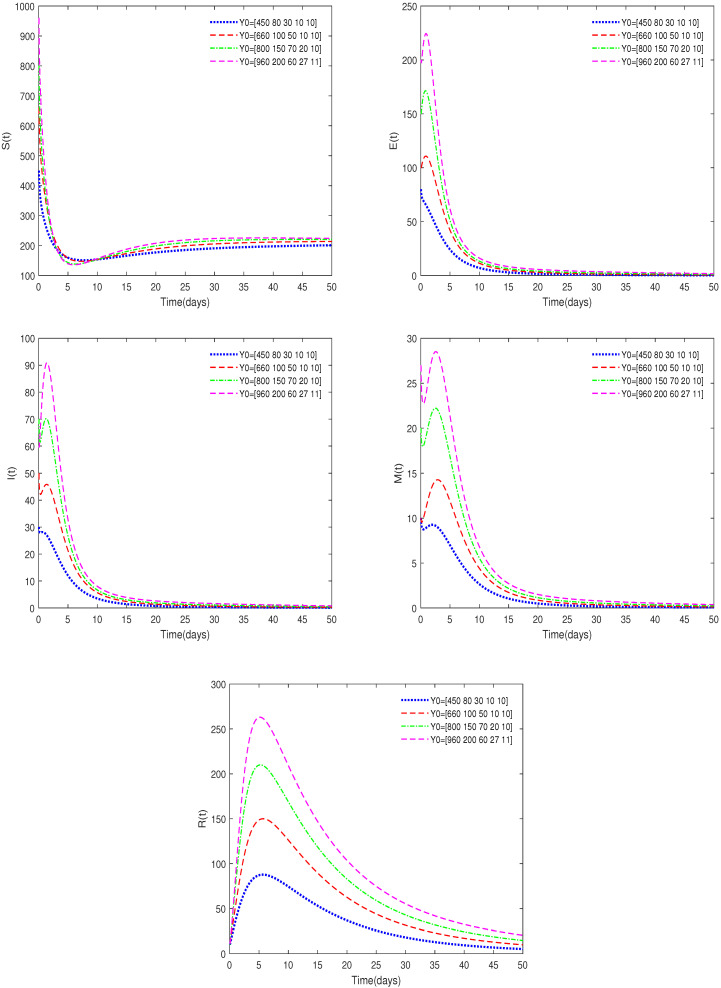
Time series of System (2) for different initial values.

*(i)*    *Figure 1 shows the variation between threshold $R_{c}$ and parameter α, with different values of φ and h (φ = 0.026, 0.057, 0.126, 0.226; h = 0.08, 0.18, 0.28, 0.38).*
*(ii)*   *In Figure 2, the initial value is $Y_{0}$ = [660, 100, 50, 10, 10], and h=0.18, φ=0.126, α have different values (α = 0.8, 0.85, 0.90, 0.93, 1). In this case, we get $R_{c}\in \left[0.8353,\;0.9314\right]<1$.*
*(iii)*  *In Figure 3, the value of α is fixed to 0.93, h=0.18, φ=0.126, and different initial values are taken with $Y_{0}$ = [450, 80, 30, 10, 10], [660, 100, 50, 10, 10], [800, 150, 70, 20, 10]. In this case, we get $R_{c}=0.8943<1$.*

**Example** **2.** *Fix the following parameter values: Λ=1.12, $\beta_{1}=0.017$, $\beta_{2}=0.0017$, μ=0.0025, δ=0.2, $b_{1}=0.007$, ω=0.12, ε=0.64, d=0.27, h=0.68, φ=0.026.*

**Figure 4:**
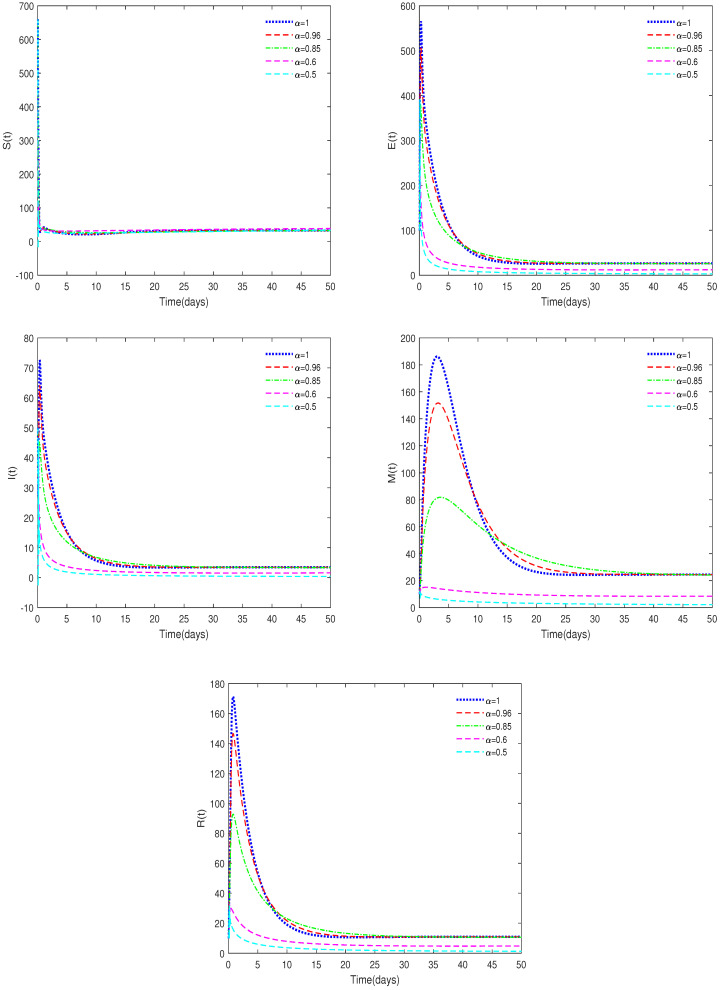
Time series of System (2) for different values of α. Here, $R_{c}\in \left[3.5421,\;16.8141\right]>$ 1.

*In Figure 4, the initial value is $Y_{0}$ = [660, 100, 50, 10, 10] and α have different values (α = 0.5, 0.6, 0.85, 0.96, 1). In this case, we get $R_{c}\in \left[3.5421,\;16.8141\right]>1$.*

**Example** **3.** *Fix the following parameter values: Λ=1.35, $\beta_{1}=0.0017$, $\beta_{2}=0.0003$, μ=0.0045, d=0.27, h=0.18, φ=0.126, α=0.93, the initial value is $Y_{0}$ = [660, 100, 50, 10, 10].*

**Figure 5:**
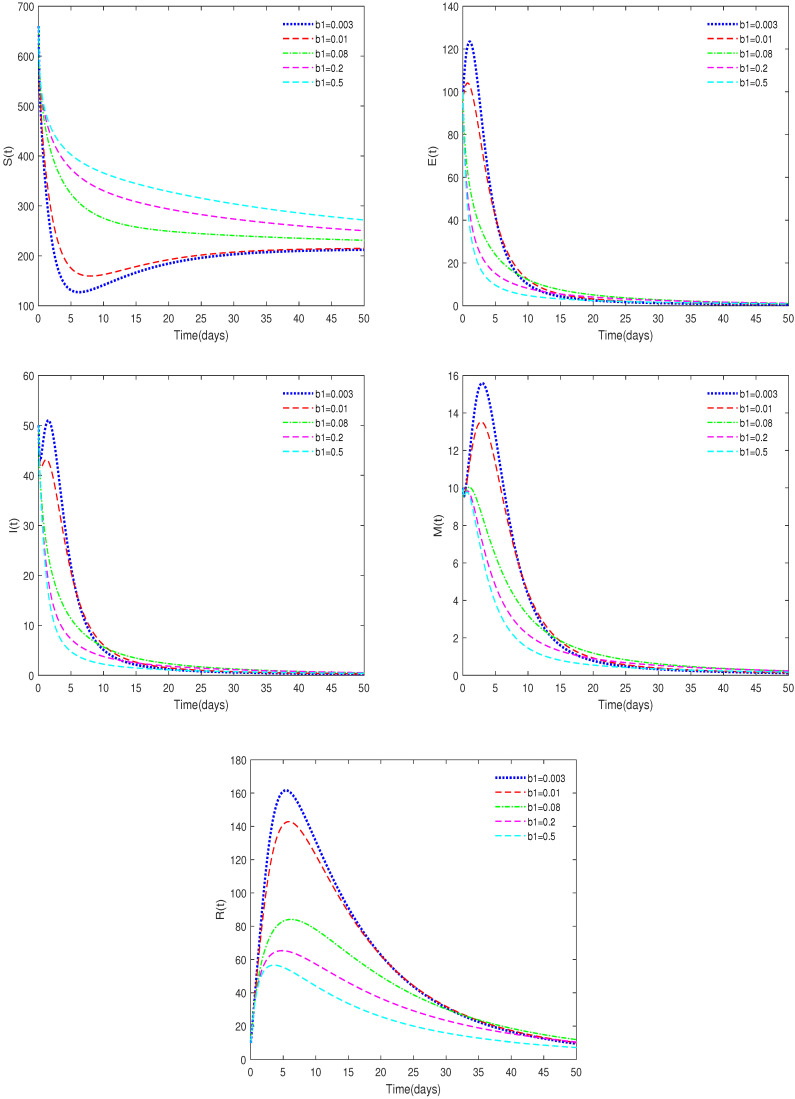
Time series of System (2) for different values of $b_{1}$.

**Figure 6:**
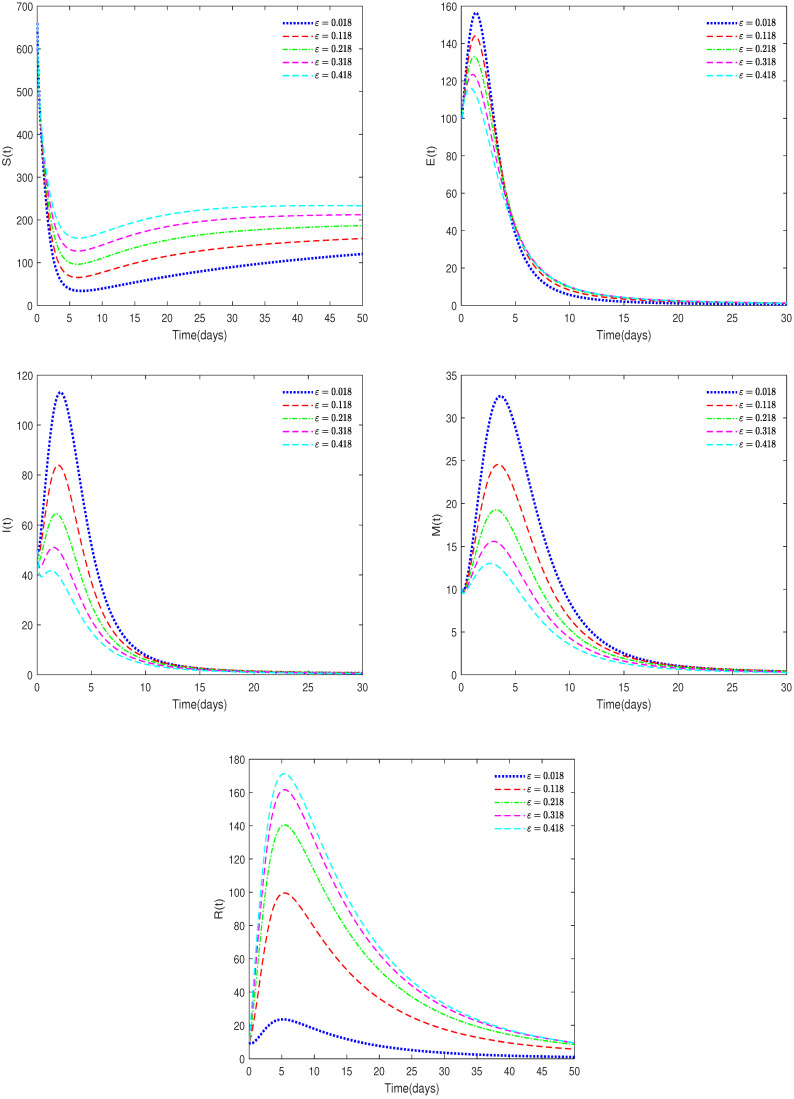
Time series of System (2) for different values of ε.

**Figure 7:**
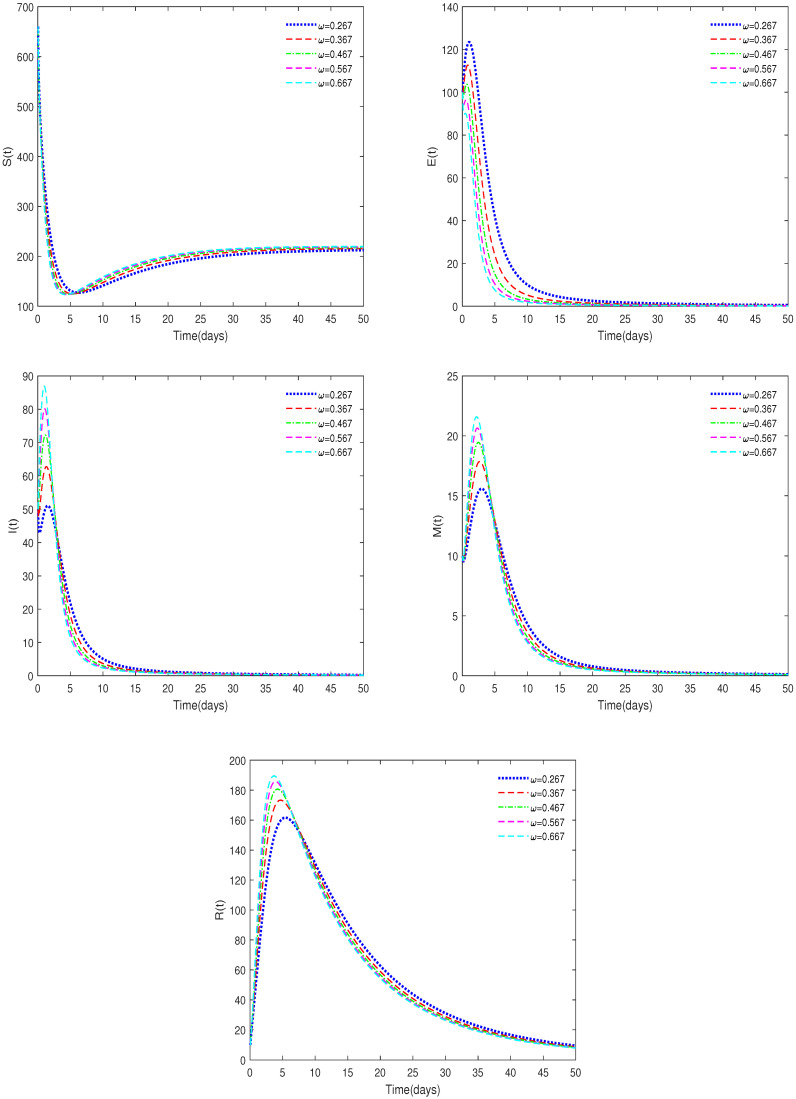
Time series of System (2) for different values of ω.

**Figure 8:**
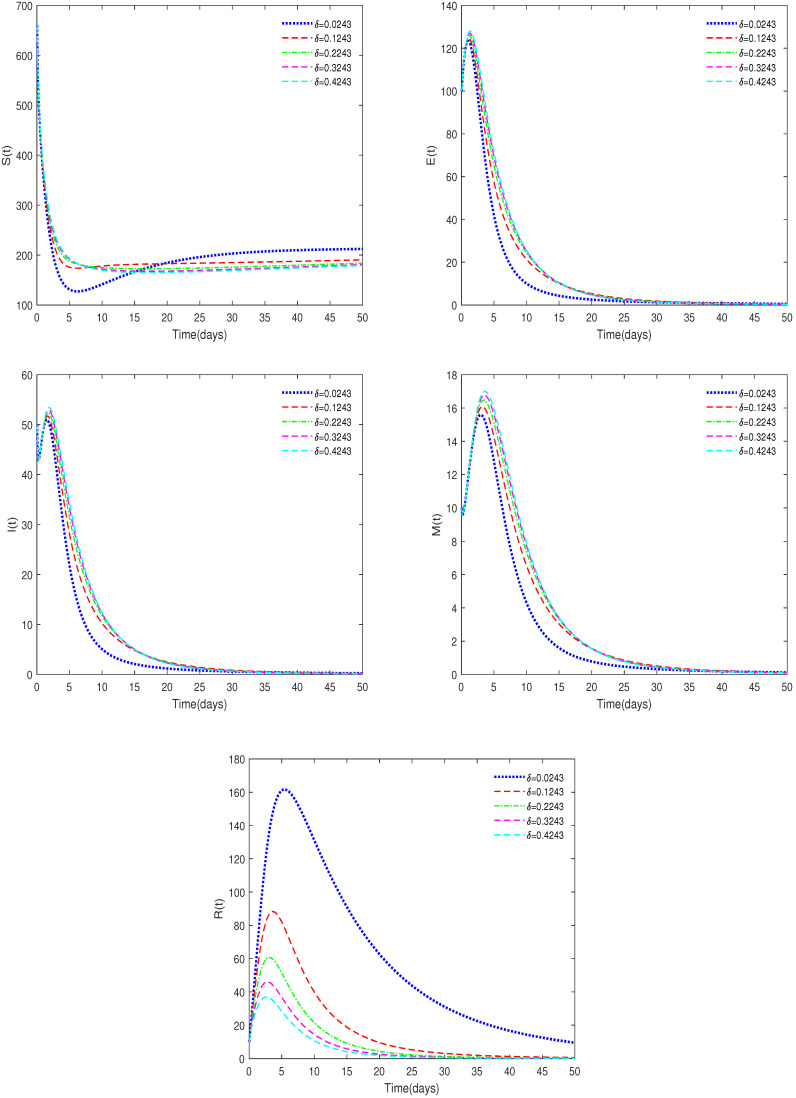
Time series of System (2) for different values of δ.

*(i)*    *In Figure 5, ε=0.3, δ=0.02, ω=0.25, and $b_{1}$ have different values ($b_{1}$ = 0.003, 0.01, 0.08, 0.2, 0.5).*
*(ii)*   *In Figure 6, $b_{1}=0.007$, δ=0.02, ω=0.25, and ε have different values (ε = 0.018, 0.118, 0.218, 0.318, 0.418).*
*(iii)*  *In Figure 7, ε=0.3, $b_{1}=0.007$, δ=0.02, and ω have different values (ω = 0.267, 0.367, 0.467, 0.567, 0.667).*
*(iv)*   *In Figure 8, ε=0.3, $b_{1}=0.007$, ω=0.25, δ=0.02, and δ have different values (δ = 0.0243, 0.1243, 0.2243, 0.3243, 0.4243).*

**Remark** **2.**

 

*(i)*    *Figure 1 shows the relationship between $R_{c}$ and the parameter α under different values of φ and h. Through observation, it can be seen that when the value of φ is relatively large, $R_{c}<1$ always holds, which means that the disease-free equilibrium, $E_{0}$, is stable. When the value of φ is relatively small, both $R_{c}>1$ or $R_{c}<1$ exist, indicating that the stability of System (2) depends on the value of α.*
*(ii)*   *From Figure 1, we can also conclude that when the value of h is relatively small, $R_{c}<1$ always holds, which means that the disease-free equilibrium, $E_{0}$, is stable. When the value of h is relatively large, both $R_{c}>1$ or $R_{c}<1$ exist, indicating that the stability of System (2) depends on the value of α.*
*(iii)*  *From a biological perspective, Figure 1 indicates that the virus release rate on diseased pigs and the virus cleaning rate in the pig breeding environment will affect the final development trend of the disease. As the cleaning rate increases, the disease will gradually move from persistence to extinction.*

**Remark** **3.**

 

*(i)*    *Figure 2 indicates that different values of α will affect the speed at which the equilibrium, $E_{0}$, approaches stability and the coordinates of $E_{0}$. When $R_{c}\in \left[0.8353,\;0.9314\right]<1$, the disease-free equilibrium, $E_{0}$, is always stable, which is in accordance with Theorems 3 and 4.*
*(ii)*  *Figure 3 indicates that the initial values will not affect the stability, which is in accordance with Theorem 1.*

**Remark** **4.** *Figure 4 indicates that different values of α will affect the speed at which the equilibrium, $E_{1}$, approaches stability and the coordinates of $E_{1}$. In this case, $R_{c}\in \left[3.5421,\;16.8141\right]>1$, but when α<0.6, the equilibrium $E_{1}$ becomes unstable, while the corresponding integer-order system remains stable, which shows the difference between fractional-order and integer-order systems. Compared to integer-order systems, the stability of fractional-order systems is more sensitive.*

**Remark** **5.**

 

*(i)*    *Figure 5 shows that the saturation constant $b_{1}$ affects the rate at which the equilibriums tend to stabilization, but does not affect the final stable state.*
*(ii)*   *Figure 6 shows that the value of parameter ε will affect the peak value of each state variable and the coordinates of the final stable state. Therefore, taking corresponding treatment measures for sick pigs is very effective for disease control.*
*(iii)*  *Figure 7 and Figure 8 show that the stability of the model does not change when some parameters fluctuate over a large range, indicating that the model is robust.*

## 5. Discussion

This article proposes a fractional-order ASF model with saturation incidence and analyzes the dynamics of the system.

The main results are as follows:

Through qualitative analysis, we get the following results:

♢ The existence and uniqueness of the positive solutions are proven and the basic reproduction numbers $R_{0}$ is obtained.
♢ The sufficient conditions for the existence and stability of disease-free equilibrium, $E_{0}$, and endemic equilibrium, $E_{1}$, are obtained.
♢ When $R_{c}<1$, the disease-free equilibrium, $E_{0}$, is globally asymptotically stable.

Through numerical simulation we get the following results.

♢ From Figure 1, we can see that the value of α has a significant impact on the threshold $R_{c}$, which in turn affects the stability of the equilibriums.
♢ Figure 1 also shows that the system is very sensitive to the values of φ and *h*. It can be seen that implementing strict cleaning measures for pig houses to reduce the virus content in the environment is an effective means of controlling the spread of the disease.
♢ Figure 2 and Figure 4 indicate that the value of α can affect the stable state of the system. For example, when $R_{c}<1$ and α<0.6, the equilibrium, $E_{1}$, is unstable, while it is stable for the corresponding integer-order system. This shows the differences between fractional-order systems and the classical integer-order systems. This also indicates that fractional-order systems have better non-locality and memory effects compared to integer-order systems.
♢ Figure 6 shows that the recovery rate parameter ε has a significant impact on the peak values of various state variables of the system. Therefore, timely treatment of sick pigs can effectively prevent healthy pigs from being infected.
♢ Compared to existing conclusions, this article focuses on discussing the difference between fractional-order systems and integer-order systems, indicating that fractional-order systems with memory characteristics are more sensitive to the dynamic of the system, and through sensitivity analysis of important parameters, effective cleaning measures have been found to have a significant impact on disease control.

## 6. Conclusions

The effective measures identified in this article are as follows:

(i)    Regular disinfection and cleaning of pigstys is essential for preventing further spread of ASF.
(ii)   The staff and external vehicles entering the pig farm should also be thoroughly disinfected to prevent the virus from being carried into the farm.
(iii)  Pigs suspected of being infected should be immediately isolated to prevent cross infection of the virus.
(iv)  The veterinarian states that pig farms should actively cooperate with local animal disease prevention and control agencies to carry out disease monitoring and investigation. The symptoms of ASF are initially very similar to those of ordinary swine fever. Therefore, when there is a failure in vaccination against swine fever or unexplained death, it is necessary to assess whether or not it is ASFV infection. The local veterinary department should be reported to in a timely manner. This is an effective means to block the further spread of ASF and reduce economic losses.

Limitations of the current work:

(i)   In this article, a deterministic model is considered. However, in the real world, the environment may be affected by some stochastic factors, so the results obtained from deterministic models may have some deviation between the model and reality. In future research, we will consider using stochastic differential equations to address current shortcomings.
(ii) This article discusses the impact of biosecurity measures on the spread of ASF. In fact, geographical factors may have a significant impact on the spread of the disease, and professional geographic information system software is available to monitor the spread of the disease [34]. This method should be applied in subsequent research.

There are still some meaningful topics to be discussed in the future:

(i)   It is well known that time delay is common in epidemic models. Thus, in the future, we can explore the cyclical impact of the incubation period of diseases on the process of disease transmission.
(ii) This article mainly discussed the stability of the system. In fact, the exact solution to the equilibrium of the system can be obtained through the Lie algebra method [35]. In future, this method will be combined to conduct more detailed research regarding the model.

## Data Availability

All data in the current study can be obtained from the corresponding author upon reasonable request.
